# Genome-Wide Gene Expression Analyses of the AtfA/AtfB-Mediated Menadione Stress Response in *Aspergillus nidulans*

**DOI:** 10.3390/cells12030463

**Published:** 2023-01-31

**Authors:** Beatrix Kocsis, Mi-Kyung Lee, Károly Antal, Jae-Hyuk Yu, István Pócsi, Éva Leiter, Tamás Emri

**Affiliations:** 1 Department of Molecular Biotechnology and Microbiology, Institute of Biotechnology, Faculty of Science and Technology, University of Debrecen, Egyetem tér 1., 4032 Debrecen, Hungary; 2 ELRN-UD Fungal Stress Biology Research Group, 4032 Debrecen, Hungary; 3 Doctoral School of Pharmaceutical Sciences, University of Debrecen, 4032 Debrecen, Hungary; 4 Biological Resource Center, Korea Research Institute of Bioscience and Biotechnology (KRIBB), Jeongeup-si 56212, Jeollabuk-do, Republic of Korea; 5 Department of Zoology, Eszterházy Károly Catholic University, Leányka Str. 6-8., 3300 Eger, Hungary; 6 Department of Bacteriology, University of Wisconsin-Madison, 1550 Linden Drive, Madison, WI 53706, USA

**Keywords:** bZIP proteins, *Aspergillus nidulans*, oxidative stress, transcriptomics

## Abstract

The bZIP transcription factors (TFs) govern regulation of development, secondary metabolism, and various stress responses in filamentous fungi. In this work, we carried out genome-wide expression studies employing Illumina RNAseq to understand the roles of the two bZIP transcription factors AtfA and AtfB in *Aspergillus nidulans*. Comparative analyses of transcriptomes of control, Δ*atfA*, Δ*atfB*, and Δ*atfA*Δ*atfB* mutant strains were performed. Dependence of a gene on AtfA (AtfB) was decided by its differential downregulation both between the reference and Δ*atfA* (Δ*atfB*) strains and between the Δ*atfB* (Δ*atfA*) and the Δ*atfA*Δ*atfB* strains in vegetatively grown cells (mycelia) and asexual spores (conidia) of menadione sodium bisulfite (MSB)-treated or untreated cultures. As AtfA is the primary bZIP TF governing stress-response in *A. nidulans*, the number of differentially expressed genes for Δ*atfA* was significantly higher than for Δ*atfB* in both mycelial and conidial samples, and most of the AtfB-dependent genes showed AtfA dependence, too. Moreover, the low number of genes depending on AtfB but not on AtfA can be a consequence of Δ*atfA* leading to downregulation of *atfB* expression. Conidial samples showed much higher abundance of *atfA* and *atfB* mRNAs and more AtfA- and AtfB-affected genes than mycelial samples. In the presence of MSB, the number of AtfB- (but not of AtfA-) affected genes decreased markedly, which was accompanied with decreased mRNA levels of *atfB* in MSB-treated mycelial (reference strain) and conidial (Δ*atfA* mutant) samples. In mycelia, the overlap between the AtfA-dependent genes in MSB-treated and in untreated samples was low, demonstrating that distinct genes can be under AtfA control under different conditions. Carbohydrate metabolism genes were enriched in the set of AtfA-dependent genes. Among them, AtfA-dependence of glycolytic genes in conidial samples was the most notable. Levels of transcripts of certain secondary metabolitic gene clusters, such as the Emericellamide cluster, also showed AtfA-dependent regulation. Genes encoding catalase and histidine-containing phosphotransfer proteins showed AtfA-dependence under all experimental conditions. There were 23 AtfB-dependent genes that did not depend on AtfA under any of our experimental conditions. These included a putative α-glucosidase (*agdB*), a putative α-amylase, *calA,* which is involved in early conidial germination, and an alternative oxidase. In summary, in *A. nidulans* there is a complex interaction between the two bZIP transcription factors, where AtfA plays the primary regulatory role.

## 1. Introduction

Basic domain leucine zipper-type (bZIP) transcription factors are members of a complex regulatory network, playing a crucial role in the maintenance and differentiation of cells as well as the coordination of stress responses in eukaryotes [1].

In filamentous fungi, the bZIP transcription factor AtfA, orthologous to Atf1 of *Schizosaccharomyces pombe* and Atf2 in mammals, orchestrates several processes, including development and secondary metabolite production of vegetative hyphae as well as stress tolerance of both vegetative hyphae and conidiospores in *A. nidulans* [2], *Claviceps purpurea* [3], *Neurospora crassa* [4], *Magnaporthe oryzae* [5], *Botrytis cinerea* [6], *Fusarium graminearum* [7], *Fusarium oxysporum* [8] and *Fusarium verticillioides* [9]. Moreover, AtfA is involved in the virulence of the human pathogenic fungi, e.g., *Aspergillus fumigatus* [10,11] and plant pathogenic fungi [3,5,6,7].

In *Aspergillus nidulans,* deletion of *atfA* resulted in oxidative, osmotic and fungicide stress sensitivity of the cultures [12,13,14,15]. The viability of the conidiospores also decreased under heat stress, in the presence of H_2_O_2_, and during storage at 4 °C in the Δ*atfA* mutant [12,13,14,15]. Microarray analysis of the Δ*atfA* mutant elucidated several stress-responsive genes likely to be regulated by AtfA, including mitotic cell cycle, nitrate reduction, tricarboxylic acid cycle, endoplasmic reticulum-related as well as FeS cluster assembly genes and elements of the two-component signal transduction system (*phkB*, *phkA*, *tcsB*, *nikA*, *hk-8-1*, *hk-8-2*, *hk-8-3*, *hk-8-4*) [16,17,18]. The global transcriptional effects of the *atfA* gene deletion were stress-type-specific and manifested mainly under menadione stress [16,17,18]. In contrast to the Δ*atfA* strain, the Δ*atfB* mutant was not sensitive to the tested oxidative stress generating agents, namely menadione sodium bisulfite (MSB), *t*-butyl-hydroperoxide or diamide; however, it was sensitive to NaCl stress [19].

In *Aspergillus oryzae,* conidia of the Δ*atfA* mutant were more sensitive to oxidative stress than those of the Δ*atfB* [20,21]. Some genes involved in the oxidative stress defense, e.g., putative catalase, thioredoxin and glutathione metabolic genes, were repressed in the Δ*atfA* mutant according to the microarray analysis, which confirms the observed stress-sensitive phenotype of the Δ*atfA* mutant [21]. In *A. oryzae, atfA* is involved in conidial storage stability [21]. Therefore, conidia of the Δ*atfA* showed lower germination rate compared to the control [21]. Most likely, *atfA* controls glutamate biosynthesis, which is necessary for germination of conidiospores [21]. *atfB* expression was significant in the late phase of culture growth and coincided with the initiation of conidiation in *A. oryzae* [21]. Furthermore, AtfB-regulated genes, such as *catA* (encoding a catalase) or a putative trehalose-6-phosphate synthase gene are most likely associated with conidial development and conidial stress tolerance [21].

bZIP transcription factors can form homodimers with themselves and heterodimers with other bZIPs and interact physically with stress-signaling proteins as well [22]. For example, in *Schizosaccharomyces pombe*, Atf1 and Pcr1 bZIPs form heterodimers and activate the majority of stress genes [23,24,25]. Evaluation of microarray data confirmed, however, that some stress genes are regulated by Atf1 independently of Pcr1 under osmotic stress (elicited with 0.4 M KCl) [24]. Moreover, Atf1 physically interacts with Cid2 poly(A) polymerase to regulate further genes [26].

In this study, we performed RNAseq-based transcriptome analysis in Δ*atfA*, Δ*atfB*, Δ*atfA*Δ*atfB* and control strains from MSB-treated and untreated surface cultures in vegetative and conidial development stages in order to understand more deeply the regulatory functions of AtfA and AtfB. We focused on the possible interactions between AtfA and AtfB during the evaluation process.

## 2. Materials and Methods

### 2.1. Strains and Culture Condition

*A. nidulans* strains (control, Δ*atfA*, Δ*atfB*, Δ*atfA*Δ*atfB)* [19] were maintained on Barratt’s nitrate minimal medium (NMM) [27], and NMM agar plates were incubated at 37 °C for 6 d [14]. Conidia harvested from these 6-day-old plates were used in all further experiments.

For RNA sequencing, freshly grown conidiospores (10^5^ suspended in 5 μL aliquots of PBS–0.1% Tween 20) were spread on NMM plates with or without 0.04 MSB (menadione sodium bisufite), and surface cultures were incubated at 37 °C. Mycelia were collected before the formation of conidiophores, while conidia were washed from the surface culture with PBS–0.1% Tween 20 and separated from the vegetative tissue with centrifugation and filtering through Miracloth.

### 2.2. RNA Sequencing

Total RNA were isolated from the menadione sodium bisulfite (MSB, a superoxide generating agent)-treated and untreated cultures of the THS30.3 (control), Δ*atfA*, Δ*atfB*, Δ*atfA*Δ*atfB* strains. Samples were taken from 20–33 hour-old (mycelial samples) and from 3-day-old (conidial samples) surface cultures of the strains. Total RNA was isolated according to Chomczynski, 1993 [28]. RNA sequencing, from library preparation to generation of fastq.gz files, was carried out at the Genomic Medicine and Bioinformatic Core Facility, Department of Biochemistry and Molecular Biology, Faculty of Medicine, University of Debrecen, Debrecen, Hungary. Libraries were prepared with the TruSeq RNA Sample preparation kit (Illumina) according to the manufacturer’s protocol. Conidial and mycelial samples were sequenced (single-read 75 bp sequencing on an Illumina HiScan SQ instrument; Illumina, San Diego, CA, USA) separately, but each library pool belonging to the same cell type was sequenced in one lane of a sequencing flow cell. Depending on the sample type, 14–39 million reads per sample (mycelial samples) and 10–28 million reads per sample (conidial samples) were obtained. The FastQC package (http://www.bioinformatics.babraham.ac.uk/projects/fastqc, accessed on 25 January 2023) was used for quality control. Reads were aligned to the genome of *A. nidulans* FGSC A4 with hisat2 (version 2.1.0) [29]. The successfully aligned reads varied between 77–96% (mycelial samples) and 78–94% (conidial samples). DESeq2 (version 1.24.0) [30] was used to determine differentially expressed genes. Since conidial samples originated in two separate experiments, the batch effect was taken into consideration during the identification of differentially expressed genes in this case. RPKM values (reads per kilo base per million mapped reads) were also calculated with the edgeR package (“rpkm” function) [31] and used to visualize transcription activities of selected genes.

### 2.3. Evaluation of the Transcriptome Data

Transcriptomes were characterized with three types of features: Strain (control, Δ*atfA*, Δ*atfB*, Δ*atfA*Δ*atfB*), treatment (untreated, MSB-treated) and cell type (mycelium, conidium). Since mycelial and conidial cultures were studied separately (e.g., the age of the studied cultures and the efficiency of RNA isolation were different), mycelial and conidial transcriptomes were not compared. We compared only transcriptomes (A vs. B) differing only in either strain or treatment. log_2_FC was calculated using the DESeq2 software, using B as reference. Differentially expressed genes were considered “upregulated” if log_2_FC > 1, “downregulated” if log_2_FC < −1 and “regulated” if |log_2_FC| > 1. “MSB stress-responsive” genes refer to genes “regulated” in an A_MSB treated_ vs. B _untreated_ comparison for any strain. The “*atfA* gene-deletion-responsive” genes are genes “regulated” in either the A_Δ*atfA*_ vs. B_control_ or A_Δ*atfA*Δ*atfB*_ vs. B_Δ*atfB*_ comparisons. The “*atfB* gene-deletion-responsive” genes were defined similarly. The *atfA atfB* gene-deletion-responsive genes were defined as genes “regulated” in the A_Δ*atfA*Δ*atfB*_ vs. B_control_ comparison.

We applied the following assumptions and simplifications during the evaluation of the data:

- Both AtfA and AtfB are positively acting transcription factors. Their target genes (AtfA-dependent genes and AtfB-dependent genes) should be downregulated in the double mutant (where both *atfA* and *atfB* were deleted).
- There are genes that are AtfA-dependent but not AtfB-dependent (AA genes) and genes that are AtfB-dependent but not AtfA-dependent (BB genes).
- There are genes regulated by both AtfA and AtfB. Some of these genes need both *atfA* and *atfB* to reach their normal physiological activity detected in the control (deletion of either gene reduces their activities at least to the level of the double mutant; AB genes). Some of the genes, regulated by both AtfA and AtfB, need either *atfA* or *atfB* for their normal activity (deletion of either gene does not reduce their transcriptional activity; A/B genes). Some of them need both *atfA* and *atfB* for normal activity and deletion of at least one of the two genes reduces the activity to a level between the control and the double mutant (A-B genes). For clarity, see Figure S1.

The following gene sets were constructed for mycelial and conidial transcriptomes:

Set0: MSB stress-responsive downregulated genes of the control strain

Set0^+^: MSB stress-responsive upregulated genes of the control strain

The following gene sets, containing downregulated genes, were also constructed for untreated and MSB-treated, mycelial and conidial transcriptomes.

From the Δ*atfA* vs. control, Δ*atfB* vs. control and Δ*atfA*Δ*atfB* vs. control comparions (Figure S1A):

Set1: Δ*atfA* vs. control (*atfA* gene-deletion-responsive downregulated genes)

Set2: Δ*atfB* vs. control (*atfB* gene-deletion-responsive downregulated genes)

Set3: Δ*atfA*Δ*atfB* vs control (*atfA atfB* double gene-deletion-responsive downregulated genes; this set contains the putative AtfA-dependent and AtfB-dependent genes)

Set4 = Set1\(Set2∪Set3)

Set5 = Set2\(Set1∪Set3)

Set6 = Set3\(Set1∪Set2) (putative A/B genes)

Set7 = (Set1∩Set2)\Set3

Set8 = (Set1∩Set3)\Set2 (putative AA and A-B genes)

Set9 = (Set2∩Set3)\Set1 (putative BB and A-B genes)

Set10 = Set1∩Set2∩Set3 (putative AB and A-B genes)

From the Δ*atfA*Δ*atfB* vs. Δ*atfB*, Δ*atfA*Δ*atfB* vs. Δ*atfA* and Δ*atfA*Δ*atfB* vs. control comparisons (Figure S1B):

Set11: Δ*atfA* Δ*atfB* vs. Δ*atfB* (*atfA* gene-deletion-responsive downregulated genes)

Set12: Δ*atfA*Δ*atfB* vs. Δ*atfA* (*atfB* gene-deletion-responsive downregulated genes)

Set13 = Set3

Set14 = Set11\(Set12∪Set13)

Set15 = Set12\(Set11∪Set13)

Set16 = Set13\(Set11∪Set12) (putative AB genes)

Set17 = (Set11∩Set12)\Set13

Set18 = (Set11∩Set13)\Set12 (putative AA and A-B genes)

Set19 = (Set12∩Set13)\Set11 (putative BB and A-B genes)

Set20 = Set11∩Set12∩Set13 (putative A/B and A-B genes)

The AA, AB, A/B and A-B genes were regarded as the intersection of the appropriate gene sets defined above:

Set21 = Set8∩Set18 (AA genes)

Set22 = Set9∩Set19 (BB genes)

Set23 = Set10∩Set16 (AB genes)

Set24 = Set20∩Set6 (A/B genes)

Set25 = (Set10∩Set20)∪(Set8∩Set20)∪(Set9∩Set20)∪(Set18∩Set10)∪(Set19∩Set10) (A-B genes; the union of the A-B1-5 gene sets, respectively)

AtfA-dependent and AtfB-dependent genes were regarded as the union of the appropriate gene sets defined above:

Set26 = Set21∪Set23∪Set24∪Set25 (AtfA-dependent genes)

Set27 = Set22∪Set23∪Set24∪Set25 (AtfB-dependent genes)

The gene sets containing the corresponding upregulated genes were marked from Set1^+^ to Set27^+^.

Note, AtfA-dependent genes (similarly to the AtfB-dependent genes) were determined from two comparisons of the four strains (Δ*atfA* vs. control and Δ*atfA-*Δ*atfB* vs. Δ*atfB* as well as Δ*atfB* vs. control and Δ*atfA-*Δ*atfB* vs. Δ*atfA*) to reduce the number of misidentified genes. These comparisons were carried out under two culturing conditions (untreated and MSB-treated cultures) in two types of cells (mycelium and conidium). This way we obtained four AtfA-dependent and four AtfB-dependent gene sets. Since AtfA- or AtfB-dependence can depend on the culturing conditions and the cell types, these gene sets were studied separately.

AtfA- and AtfB-dependent gene sets were characterized by gene set enrichment analyses. For it, “Functional Catalogue” (FunCat), “Gene Ontology” (GO) and “Kyoto Encyclopedia of Genes and Genomes pathway” (KEGG pathway) terms were used with the FungiFun2 package (https://elbe.hki-jena.de/fungifun/fungifun.php, accessed on 25 January 2023) applying default settings. Only hits with a corrected *p*-value < 0.05 were regarded as significantly enriched in the studied gene set.

The enrichment of the following gene groups in the AtfA- and AtfB-dependent gene sets were also tested by the Fisher’s exact test with the “fisher.test” function of R project (www.R-project.org/, accessed on 25 January 2023):

“Lactose utilization” genes. This gene group contains the Leloir and oxido-reductive pathways of galactose utilization [32] as well as known and putative β-galactosidase and lactose permease genes according to Fekete et al. 2012, 2016 [33,34] and Gila et al. 2022 [35].

“Antioxidant enzyme” genes. Genes of known, or putative superoxide dismutases, catalases, peroxidases, and the glutathione/glutaredoxin/thioredoxin redox system according to Gila et al. 2021 [36].

“Glycolysis” genes, “Oxidative pentose-phosphate shunt” genes, “Ribose metabolism” genes and “TCA cycle” genes. Genes described by Flipphi et al. 2009 [37].

“Carbohydrate-active enzyme” (CAZyme) genes. Genes collected from the Carbohydrate-active Enzymes Database (http://www.cazy.org, accessed on 25 January 2023).

Phosphorelay response regulator activity, iron-sulfur cluster assembly, “Respiration”, and “Transcription factor” genes. These groups were constructed based on the related GO terms and their child terms [35,36].

“Secondary metabolism cluster” genes. Manually or experimentally determined secondary metabolite cluster genes collected by Inglis et al. 2013 [38] and gene set enrichment analysis was carried out with the clusters separately.

## 3. Results

### 3.1. Deletion of atfA Downregulates atfB

Mycelial and conidial transcriptomes from four strains (control, Δ*atfA*, Δ*atfB*, Δ*atfA* Δ*atfB*) at two different culturing conditions (untreated, MSB-treated) were determined. Changes in either feature (strain, treatment) had substantial effects on the transcriptomes (Figure S2). Genes responsive for gene deletions (in untreated and MSB-treated cultures), for MSB treatment (in the control strain) or that showed AtfA- and/or AtfB-dependence were identified in both mycelial and conidial samples (Table 1, Table 2 and Table S1).

**Figure 1:**
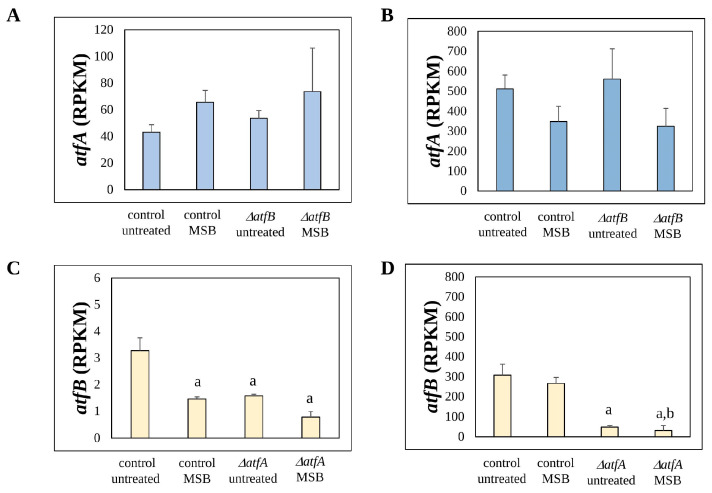
Transcriptional profile of the *atfA* and *atfB* genes. (**A**,**B**): Expression of *atfA* in MSB treated and untreated cultures of the wild type and the Δ*atfB* strain. (**C**,**D**): Expression of *atfB* in MSB treated and untreated cultures of the wild type and the Δ*atfA* strain. Mean ± SD RPKM values calculated from three biological replicates of mycelial (**A**,**C**) and conidial (**B**,**D**) samples are presented. a—downregulated gene relative to the untreated control cultures, b—downregulated gene relative to the MSB treated control cultures.

MSB treatment and deletion of the *atfB* gene did not upregulate or downregulate the *atfA* gene in *atfA^+^* strains (Figure 1). In contrast, the presence of MSB (in the case of mycelial samples) and the deletion of *atfA* (in both mycelial and conidial samples) downregulated the *atfB* gene (Figure 1). This means that AtfA can affect the transcription of AtfB-dependent genes via *atfB* transcription; therefore, some of the genes putatively regulated by both AtfA and AtfB may be genes that were regulated directly only by AtfB. These genes can potentially occur in any gene sets but especially in those where the effect of *atfB* deletion was stronger than or equal with that of the *atfA* gene deletion: AB (Set23), A-B1 and A-B5 (Set25) gene sets (Table 1 and Table 2). Importantly, no genes belonging to the A-B5 (Set25) gene set were identified (Table 1 and Table 2).

### 3.2. Most of the AtfB-Dependent Genes Show AtfA-Dependence in Mycelia of Untreated Cultures

Many more *atfA* gene-deletion-responsive genes were found in untreated mycelial samples than *atfB* gene-deletion-responsive genes (Table 1). The difference between the Δ*atfA* and the Δ*atfB* strains was more obvious in the case of the downregulated genes than with the upregulated ones (Table 1). Altogether, 329 AtfA- and 96 AtfB-dependent genes were identified in these cultures, and most of the AtfB-dependent genes showed AtfA-dependence as well (Table 1). The high number of AtfA-dependent genes relative to the number of AtfB-dependent genes concurs well with *atfA* gene deletion having stronger transcriptomical (Figure S2, Table 1) and physiological [19] consequences than *atfB* gene deletion.

**Figure 2:**
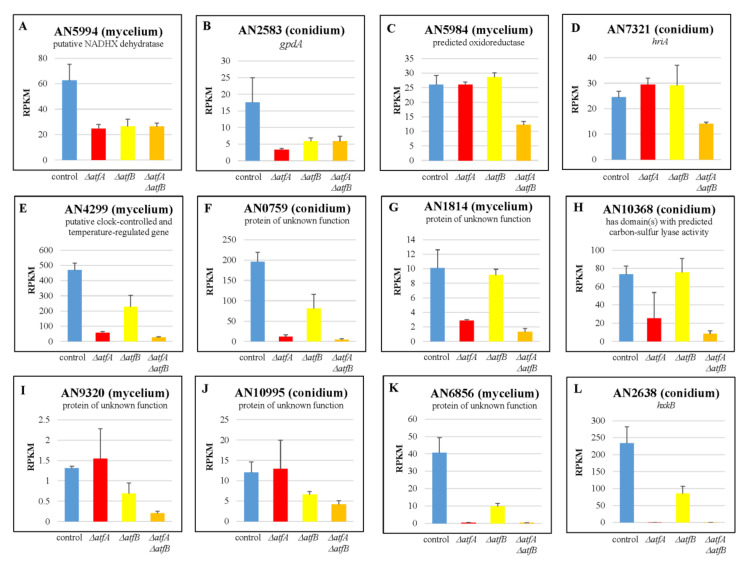
Transcriptional pattern of selected genes potentially regulated by both AtfA and AtfB. (**A**,**B**): AN5994 and AN2583 (AB genes; Set 23), (**C**,**D**): AN5984 and AN7321 (A/B genes; Set24), (**E**,**F**): AN4299 and AN0759 (A-B1 genes; Set25), (**G**,**H**): AN1814 and AN10368 (A-B2 genes; Set25, (**I**,**J**): AN9320 and AN10995 (A-B3 genes; Set25), (**K**,**L**): AN6856 and AN2638 (A-B4 genes; Set25) (see also Figure S2). Mean ± SD of the three biological replicates from mycelial (**A**,**C**,**E**,**G**,**I**,**K**) and conidial (**B**,**D**,**F**,**H**,**J**,**L**) samples of untreated (**A**–**D**,**F**–**L**) and MSB-treated (**E**) cultures are presented.

According to the type of possible interactions between the regulatory effects of AtfA and AtfB, genes that showed both AtfA- and AtfB-dependence were grouped into three sets: AB, A/B, and A-B (Figure 2, Table 1). The most interesting group was the AB set. The transcriptional pattern of the related genes (Figure 2, Table S2) suggests that both AtfA and AtfB were needed for their normal (“wild type”) activity. Besides the genes regulated by AtfA via regulation of the *atfB* gene, it is possible that some of these genes were regulated by an AtfA-AtfB heterodimer. The majority of the genes under both AtfA and AtfB regulations belonged to the A/B or A-B gene sets (Table 1 and Table S2), suggesting that the missing transcription factor was completely (A/B) or at least partially (A-B) substituted with the other transcription factor. In the case of the most A-B genes, *atfA* gene deletion had a stronger consequence than that of *atfB* (A-B2 and A-B4 genes) (Figure 2, Table S2).

### 3.3. AtfB Regulates Only Few Genes in Mycelia of MSB-Treated Cultures

The transcription of fewer genes was affected by *atfA* and/or *atfB* gene deletions on MSB than in untreated cultures (Table 1). Only a few AtfB-dependent genes (10 genes) were found in this case, and most of these genes were AtfA-dependent too (Table 1). This concurs well with the observation that *atfB* was downregulated by MSB stress in the control strain (Figure 1). Not surprisingly, the Δ*atfB* mutant was as sensitive to MSB stress as the control strain [19].

The overlaps between the MSB-treated and untreated cultures in the cases of the gene-deletion-responsive gene sets and the AtfA-dependent gene sets were relatively small (Table 1), supporting the view that AtfA regulates (directly or indirectly) different genes in mycelia under different culturing conditions [16,17,18]. Surprisingly, the AtfA-dependent gene set of MSB-treated cultures, similarly to the AtfA and AtfB-dependent gene sets of untreated cultures, was enriched in MSB stress-responsive downregulated genes (Table 1), i.e., many genes that were downregulated by the presence of MSB showed further downregulation in the absence of AtfA. This behavior suggests that one of the main functions of AtfA in cultures that have adapted to the presence of MSB is not to keep high the transcriptional activity of genes upregulated by the stress treatment but to prevent the excessive downregulation of genes downregulated under this stress. Of course, this does not exclude that several genes are upregulated by AtfA during the early stress response of MSB stress.

### 3.4. AtfB, Similarly to AtfA, Increases Importance in Conidia

The transcriptional activity of both *atfA* and *atfB* was much higher in conidia than in mycelial samples (Figure 1). Not surprisingly, huge numbers of *atfA* and/or *atfB* gene-deletion-responsive and AtfA- and/or AtfB-dependent (Table 2) genes were recorded in conidial samples, demonstrating that AtfA and AtfB are more important regulators of the physiology of (germinating) conidia than of the vegetative mycelia. MSB treatment did not reduce the abundance of *atfB* transcript in conidia (Figure 1); however, the number of AtfB-dependent genes decreased more radically in the presence of MSB than did AtfA-dependent genes (Table 2).

Again, most of the AtfB-dependent genes showed AtfA-dependence as well (Table 2 and Table S2). Among the genes showing both AtfA- and AtfB-dependence, the AB, A/B, A-B2 and A-B4 genes were the most abundant (Table 2 and Table S2). Accordingly, AtfB regulates only few genes independently of AtfA (BB genes, Table 2 and Table S1). Some genes are regulated together with AtfA (AB genes, Table 2), and in the case of the most AtfB-dependent genes AtfA can replace the missing AtfB (A/B, A-B2, A-B4 genes, Table 2 and Table S2).

MSB treatment substantially modified the transcriptome of both conidia and mycelia: 485 and 786 upregulated and 1070 and 912 downregulated MSB stress-responsive genes were found in conidial and mycelial samples, respectively. The effect of MSB treatment on the conidial transcriptome suggests that stresses affecting the physiology of mycelia also affect the transcriptome of conidia produced by the stress-treated mycelia. Interestingly, these changes modified only slightly the regulatory role of AtfA and AtfB in conidia: The overlaps between the AtfA- (AtfB-) dependent genes of conidia from untreated and MSB-treated cultures were large, in contrast to those in mycelial samples (Table 1 and Table 2). It can be understandable if we assume that the transcriptional changes of mycelia reflect how vegetative cells adapted to the presence of MSB, while the transcriptional changes in conidia show how the “experiences” of mycelia are implemented into the germination strategies of conidia. In other words, conidia do not have to adapt to all consequences of long-term MSB treatment; they have to prepare only for the increased possibility of MSB stress during their germination.

Gene sets identified with conidial samples showed surprisingly low overlap with the appropriate mycelial gene sets (Table 2). Even in the case of the AtfA-dependent genes, the overlap was only around 50% (Table 2). This huge difference between mycelial and conidial samples suggests that AtfA and AtfB had different functions and that *atfA* and *atfB* gene deletions had different consequences in mycelia and in conidia.

### 3.5. AtfA Affects Carbohydrate Metabolism and Light Dependent Processes

Gene set enrichment analyses were carried out with four AtfA- and four AtfB-dependent gene sets (identified in untreated mycelial and conidial samples as well as in MSB-treated mycelial and conidial samples) (Table 3, Table 4, Tables S3 and S4).

The AtfA-dependent gene sets were enriched with carbohydrate metabolism genes. Among them, AtfA-dependence of glycolytic genes in the case of the conidial samples is the most notable (Table S3). Phosphorelay response regulator genes were enriched in all AtfA-dependent gene sets but the untreated mycelial samples (Table 4 and Table S4), while enrichment of iron-sulfur cluster assembly genes was characteristic for the AtfA-dependent genes of conidial samples (Table 4 and Table S4). Interestingly, antioxidant enzyme genes were enriched only in the AtfA-dependent gene set of conidial samples from untreated cultures (Table 4 and Table S4). Enrichment of TCA cycle and respiration genes was characteristic for the AtfA-dependent gene set of conidial samples from MSB-treated cultures. Most of the above-mentioned genes were regulated only by AtfA (AA genes) (Table S4). Certain secondary metabolite cluster genes also showed AtfA-dependent regulation (Table 4 and Table S4). Among them, the Emericellamide cluster is notable since, depending on the treatment, four or five genes out of the five cluster genes were AtfA-dependent, including the *easB* gene (AN2547) encoding the polyketide synthase (Figure S3, Table S3). Interestingly, in untreated cultures, these genes showed both AtfA- and AtfB-dependence (A/B genes), while in MSB-treated cultures, where *atfB* was downregulated (Figure 1), they were only AtfA-dependent (AA genes).

Altogether 87 genes showed AtfA-dependence in all the four AtfA-dependent gene sets. Most of them encode proteins with unknown functions (Table 5). The genes with known or predicted function includes the *catA* catalase, six genes involved in carbohydrate metabolism as well as the *hk-8-1* and *hk2* putative histidine-containing phosphotransfer protein genes and 10 genes involved in light sensing and light response (Tables S1 and S4).

Only 23 AtfB-dependent genes were found that never showed AtfA-dependence (Table S1). Out of them, the following four genes are notable: AN8953 (*agdB*), putative α-glucosidase and AN3402 (*amyB*), putative α-amylase genes; AN7619 (*calA*), involved in early conidial germination; and AN2099, putatively encoding alternative oxidase.

## 4. Discussion

bZIP-type transcription factors are important regulators of developmental processes, stress responses and secondary metabolite production in filamentous fungi [19,39,40,41,42]. They can act as homodimers, and they can also regulate processes forming heterodimers with other bZIP-type transcription factors or physically interact with other signaling proteins [22]. In *A. fumigatus*, AtfA physically interacts with other three bZIP transcription factors, namely AtfB, AtfC and AtfD, as well as with the MAPK SakA to coordinate stress responses [11]. According to the stress sensitivity assays, the Δ*atfA*Δ*atfB* double-gene deletion mutant was as sensitive to the oxidative stress generating menadione sodium bisulfite or to the cell wall stress-generating agents calcofluor white (CFW) and CongoRed as the corresponding single mutants in *A. fumigatus* [11]. In *A. parasiticus*, AtfB and AP-1 bZIPs form functionally active heterodimers and regulate aflatoxin production and oxidative stress responses [39]. In the case of *A. nidulans,* Lara-Rojas et al. 2011 [2] suggested possible physical interaction between AtfA and AtfB. Here we studied genome-wide transcriptional changes in mycelia and conidia of Δ*atfA*, Δ*atfB*, Δ*atfA*Δ*atfB* gene-deletion mutants and the control strain in the presence and absence of MSB to collect data on the possible interactions between AtfA and AtfB.

The high transcriptional activity of *atfA* (Figure 1) and the genome-wide transcriptional as well as phenotypic consequences of *atfA* gene deletion (Table 1 and Table 2; [15,19]) suggest the importance of AtfA-dependent regulations in both mycelia and conidia. The data also support the view that AtfA was a more important regulator in conidia than in vegetative mycelia (Figure 1, Table 1 and Table 2). This concurs well with the observations of Hagiwara et al. 2008 [12] and Balázs et al. 2010 [14] that AtfA protects conidia under different temperatures as well as against oxidative stress. AtfA plays a paramount role in the regulation of conidium-specific genes in other *Aspergillus* species as well. In a comprehensive study, more than 50% of the conidia-associated genes (CAGs) proved to be *atfA*-dependent in *A. fumigatus*, *A. oryzae* and *A. niger* [43].

Many genes regulated (directly or indirectly) by AtfA have been identified so far in *Aspergillus* species. These genes–among others–encode antioxidant proteins, heat shock proteins, phosphorelay response regulators, iron-sulfur cluster assembly proteins, enzymes involved in trehalose and glycogen formation, utilization of different carbohydrates or secondary metabolite synthesis as well as light response of conidia [11,16,17,18,21,43,44,45,46,47]. Our data support the role of AtfA in the transcription of antioxidant enzyme and phosphorelay response regulator genes, trehalose and glycogen metabolism genes, glucose utilization genes as well as secondary metabolite cluster genes (Table 3, Table 4 and Table S4). The cytoplasmic phytochrome FphA (acting as red-light sensor) activates AtfA via the high-osmolarity glycerol (HOG) MAPK pathway in *A. nidulans* [46,47]. Not surprisingly, several light-dependent genes were identified as AtfA-dependent in our study (Table 5 and Table S1). Among them, *cryA* encoding a putative UV-A/blue light sensor (cryptochrome) [48] is particularly interesting. Yu et al. 2016 [46] found that the blue-light-dependent activation of the HOG pathway depends on FphA only, but not on the blue-light sensor LreA-LreB complex. One explanation of this observation is that another blue-light receptor (e.g., CryA) is involved in this process and its activity is somehow regulated by FphA [46]. The AtfA-dependent transcription of *cryA* (Table 5 and Table S1) also supports the view that there is interaction between the blue- and red-light dependent signaling pathways.

Stress tolerance of conidia highly depends on culturing conditions occurring during conidiogenesis [49,50,51,52]. Not surprisingly, MSB stress treatment (of mycelia) affected both the mycelial and conidial transcriptomes (Table 1 and Table 2). The regulatory role of AtfA also depended on culturing conditions: Different genes showed AtfA-dependence in MSB stress-adapted and unstressed cultures (Table 1 and Table 2). Importantly, the difference between the AtfA-dependent gene sets was more obvious in mycelial than in conidial samples (Table 1 and Table 2). It is reasonable to assume that conidia, de facto, do not have to adapt to the presence of MSB. Conidiogen cells alter the mRNA content of conidia only to prepare them for the stresses that (according to their “experiences”) most likely will occur during germination.

Transcriptional activity of *atfB* was low in mycelial samples; however, *atfB* mRNA was abundant in conidia (Figure 1), suggesting that this transcription factor may have a minor regulatory role during vegetative growth. The small transcriptomic (Table 1 and Table 2) and phenotypic [19] consequences of *atfB* gene deletion relative to that of *atfA* also support this view.

The majority of the AtfB-dependent genes were AtfA-dependent as well (Figure 2, Table 1, Table 2 and Table S2), which concurs well with results of Sakamoto et al. 2009 [21], who also found that most of the stress-responsive genes regulated by AtfB were also AtfA-dependent in *A. oryzae*. Some of the genes that showed dual AtfA- and AtfB-dependent regulation needed both AtfA and AtfB for their “normal” expression (AB genes on Figure 2 and in Table 1, Table 2 and Table S2). It is possible that some of them are regulated by an AtfA–AtfB heterodimer; however, without experimental justification, other possibilities cannot be ruled out. The majority of the AtfA-, AtfB-dependent genes were genes where one of the two transcription factors could completely or partially substitute the missing other transcription factor (A/B and A-B genes on Figure 2 and in Table 1, Table 2 and Table S2). Some of these genes may be regulated by both transcription factors directly, which also allows physical interaction between the two transcription factors on the promoters. Moreover, *atfB* itself also showed AtfA-dependence; deletion of *atfA* downregulated *atfB* in both mycelial and conidial samples irrespectively of the presence of MSB (Figure 1). Therefore, some of the genes showing both AtfA- and AtfB-dependence can be AtfB-dependent genes regulated by AtfA only indirectly via *atfB* transcription. The interaction between AtfA and AtfB has also been suggested by the overexpression of *atfB* being able to compensate for the increased MSB sensitivity of the Δ*atfA* mutant [19]. Moreover, sterigmatocystin production was completely inhibited by *atfA* gene deletion; however, it was restored in the Δ*atfA*Δ*atfB* mutant [19]. Importantly, we found a few AtfB-dependent genes that did not show AtfA-dependence (Table 1, Table 2, Tables S1 and S4). Among them, *calA* is notable, since it contributes to the germination of conidia [53,54] which may explain why conidia of the Δ*atfB* strain were sensitive to high temperature [19].

Our results support the view that (1) AtfA and AtfB have some regulatory functions in mycelia; however, they are more important regulators in conidia. (2) Besides regulating antioxidant enzyme genes, phosphorelay response regulator genes, secondary metabolite cluster genes, and light-dependent genes, AtfA also control genes of carbohydrate metabolism (e.g., trehalose and glycogen metabolism genes as well as glucose utilization genes) in *A. nidulans,* as it was also found in *A. fumigatus* [11]. (3) There should be a complex genetic and possibly physical interaction between the two transcription factors where AtfA is the dominant player, and the main function of AtfB is supporting the regulatory role of AtfA. Understanding the nature of the interaction between the two transcription factors needs further investigations: e.g., determining the AtfA- and AtfB-binding sites on the promoters at genome level by combining chromatin immunoprecipitation assays with sequencing (ChIP-Seq), and justifying the AtfA–AtfB heterodimer formation using a bimolecular fluorescence complementation (BiFC) technique. Both are in progress in our laboratory.

## Figures and Tables

**Table 1:** Characteristics of the main gene sets identified with mycelial samples.

Gene Set	Mycelium from Untreated Cultures	Mycelium from MSB-Treated Cultures	Overlap between MSB-Treated and Untreated Cultures
Responsive to *atfA* gene deletion (Set1 and Set1^+^)	326 upregulated genes 865 downregulated genes	255 upregulated genes 583 downregulated genes	85 upregulated genes 336 downregulated genes
Responsive to *atfB* gene deletion (Set2 and Set2^+^)	213 upregulated genes 159 downregulated genes	201 upregulated genes 77 downregulated genes	71 upregulated genes 12 downregulated genes
Responsive to *atfA atfB* double-gene deletion (Set3 and Set3^+^)	200 upregulated genes 457 downregulated genes	131 upregulated genes 296 downregulated genes	45 upregulated genes 142 downregulated genes
AtfA-dependent genes ^a^ (Set26)	329 genes (236 AA, 16 AB, 40 A/B, 1 A-B1, 3 A-B2 and 33 A-B4 genes) (10 upregulated and 218 ^b^ downregulated MSB stress-responsive genes)	240 genes (232 AA, 2 AB, 4 A/B and 2 A-B3 genes) (17 upregulated and 143 ^b^ downregulated MSB stress-responsive genes)	110 genes
AtfB-dependent genes (Set27)	96 genes (3 BB, 16 AB, 40 A/B, 1 A-B1, 3 A-B2 and 33 A-B4 genes) (2 upregulated and 68 ^b^ downregulated MSB stress-responsive genes)	9 genes (1 BB, 2 AB, 4 A/B and 2 A-B3 genes) (1 upregulated and 3 downregulated MSB stress-responsive genes)	3 genes

^a^—The lists of AtfA- and AtfB-dependent genes are available in Table S1. ^b^—Significant enrichment (Fisher’s exact test, *p* < 0.05). MSB stress treatment upregulated 786 genes and downregulated 912 genes (Set0^+^ and Set0, respectively).

**Table 2:** Characteristics of the main gene sets identified with conidial samples.

Gene Set ^a^	Conidium from Untreated Cultures	Conidium from MSB-Treated Cultures	Overlap between MSB-Treated and Untreated Cultures
Responsive to *atfA* gene deletion (Set1 and Set1^+^)	1875 upregulated genes (82; 25% ^c^) 2116 downregulated genes (326; 38%)	1386 upregulated genes (57; 22%) 1480 downregulated genes (167; 29%)	902 upregulated genes 1274 downregulated genes
Responsive to *atfB* gene deletion (Set2 and Set2^+^)	117 upregulated genes (5; 2%) 396 downregulated genes (36; 23%)	74 upregulated genes (7; 3%) 161 downregulated genes (1; 1%)	30 upregulated genes 108 downregulated genes
Responsive to *atfA atfB* double-gene deletion (Set3 and Set3^+^)	1604 upregulated genes (32; 16%) 2018 downregulated genes (248; 54%)	1553 upregulated genes (27; 21%) 1547 downregulated genes (125; 42%)	848 upregulated genes 1374 downregulated genes
AtfA-dependent genes (Set26)	1496 genes (185; 56%) (1079 AA, 80 AB, 84 A/B, 13 A-B1, 75 A-B2, 4 A-B3 and 161 A-B4 genes) (6 upregulated and 319^b^ downregulated MSB stress-responsive genes)	1143 genes (105; 44%) (989 AA, 41 AB, 8 A/B, 2 A-B1, 12 A-B2 and 91 A-B4 genes) (21 upregulated and 154 ^b^ downregulated MSB stress-responsive genes)	1043 genes
AtfB-dependent genes (Set27)	439 genes (26; 27%) (22 BB, 80 AB, 84 A/B, 13 A-B1, 75 A-B2, 4 A-B3 and 161 A-B4 genes) (1 upregulated and 178 ^b^ downregulated MSB stress-responsive genes)	155 genes (1; 11%) (1 BB, 41 AB, 8 A/B, 2 A-B1, 12 A-B2 and 91 A-B4 genes) (5 upregulated and 22 downregulated MSB stress-responsive genes)	114 genes

^a^—The lists of genes belonging to the sets are available in Table S1. ^b^—Significant enrichment (Fisher’s exact test, *p* < 0.05). MSB stress treatment upregulated 485 genes and downregulated 1070 genes (Set0^+^ and Set0, respectively). ^c^—Figures in parentheses shows the overlap between conidial and mycelial samples as well as the percentage of overlap relative to the size of the mycelial set. In the case of the mycelial and conidial Set0^+^ and Set0 gene sets, the overlaps were 45 upregulated and 225 downregulated genes, which represent 6% and 25% of the mycelial gene sets, respectively.

**Table 3:** Selected significantly enriched FunCat, GO and KEGG pathway term of the AtfA- and AtfB-dependent gene sets. The full list of significantly enriched terms is available in Table S2.

Culture	AtfA-Dependent Genes (Set26)	AtfB-Dependent Genes (Set27)
Mycelium (untreated)	C-compound and carbohydrate metabolism; galactose metabolic process	
Mycelium (MSB-treated)	Amine/polyamine transport	
Conidium (untreated)	C-compound and carbohydrate metabolism; C-compound and carbohydrate transport; glycolysis and gluconeogenesis; pentose phosphate pathway; fructose and mannose metabolism; pyruvate metabolism; glyoxylate and dicarboxylate metabolism; homeostasis of phosphate; proton-driven antiporter; sodium-driven symporter; biosynthesis of secondary metabolites; cellular sensing and response to external stimulus; oxidative stress response	C-compound and carbohydrate metabolism; C-compound and carbohydrate transport; glycolysis and gluconeogenesis; starch and sucrose metabolism; valine, leucine and isoleucine degradation; biosynthesis of secondary metabolites;
Conidium (MSB-treated)	C-compound and carbohydrate metabolism; glycolysis and gluconeogenesis; pentose phosphate pathway; fructose and mannose metabolism; glyoxylate and dicarboxylate metabolism; TCA cycle; homeostasis of phosphate; biosynthesis of secondary metabolites; cellular sensing and response to external stimulus; peroxisome	

**Table 4:** Selected gene groups significantly enriched (Fisher’s exact test; *p* < 0.05) in the AtfA- and AtfB-dependent gene sets. The full list is available in Table S3.

Culture	AtfA-Dependent Genes (Set26)	AtfB-Dependent Genes (Set27)
Mycelium (untreated)	Secondary metabolism: No PKS/NRPS backbone cluster 1, Microperfuranone cluster, AN2924 cluster; AN9005 cluster; AN10297 cluster; Emericellamide cluster	Secondary metabolism: AN2924 cluster; AN10297 cluster; Emericellamide (eas) cluster
Mycelium (MSB-treated)	CAZyme genes Phosphorelay response regulator genes Secondary metabolism: Aspercryptin cluster, AN2924 cluster; AN10297 cluster; Emericellamide (eas) cluster	
Conidium (untreated)	Glycolysis; Pentose-phosphate shunt; Leloir pathway Antioxidative enzyme genes; iron-sulfur cluster assembly Phosphorelay response regulator genes Secondary metabolism: AN9005 cluster; AN1594 cluster; AN10297 cluster; AN1242 cluster	Transcription factors Secondary metabolism: AN9005 cluster; AN10297 cluster
Conidium (MSB-treated)	Glycolysis; pentose-phosphate shunt; Leloir pathway; TCA cycle; respiration Iron-sulfur cluster assembly Phosphorelay response regulator genes Secondary metabolism: AN1594 cluster; AN10297 cluster; AN1242 cluster	

**Table 5:** Selected genes showing AtfA-dependent in all cultures.

Gene ID	Gene Name	Description	AtfA/AtfB-Dependence in			
			Mycelium (Untreated)	Mycelium (MSB-Treated)	Conidium (Untreated)	Conidium (MSB-Treated)
Light dependent regulation						
AN0387	*cryA*	senses UVA and blue light	AA	AA	AA	AA
AN5056		induced by light	A-B	AA	AA	AA
AN9285	*ccgA*	induced by light	AA	AA	AA	A-B
AN4299		induced by light	A-B	AA	AA	AA
AN8638	*cetJ*	induced by light	AA	AA	AA	AA
AN0045		induced by light	A-B	AA	AA	AA
AN0693		induced by light	AA	AA	AA	AA
AN5004		induced by light	A-B	AA	A-B	AA
AN8339		induced by light	AA	AA	AA	AA
AN8641		induced by light	A-B	AA	A-B	AA
Carbohydrate metabolism						
AN8138	*aglC*	α-galactosidase	A-B	AA	A-B	AA
AN2835		predicted D-arabinono-1,4-lactone oxidase activity	AA	AA	A-B	AA
AN8639		putative α,α-trehalose-phosphate synthase	AA	AA	A-B	A-B
AN10060		putative α-amylase	AA	AA	AA	AA
AN3200		putative β-glucuronidase	AA	AA	AA	AA
AN9180		putative transketolase	AA	AA	AA	AA
Other						
AN2470		cellular response to nitrosative stress	AA	AA	AA	AA
AN8637	*catA*	conidia-specific catalase	AA	AA	AA	AA
AN2581	*hk-8-1*	putative histidine-containing phosphotransfer protein	AA	AA	AA	AA
AN7945	*hk2*	putative histidine-containing phosphotransfer protein	AA	AA	AA	AA
AN9005		putative polyketide synthase	AA	AA	AB	AB

AA—regulated putatively by AtfA but not by AtfB. AB—Regulated putatively by both AtfA and AtfB; deletion of either *atfA* or *atfB* reduces the gene activity to the level of the double mutant. A-B—Regulated putatively by both AtfA and AtfB; deletion of *atfA* and/or *atfB* reduces only partially the gene activity.

## Data Availability

Transcriptome data sets are available with the GSE220052 accession number of the Gene Expression Omnibus database (GEO; http://www.ncbi.nlm.nih.gov/geo/, accessed on 25 January 2023).

## Supplementary Materials

The following supporting information can be downloaded at: https://www.mdpi.com/article/10.3390/cells12030463/s1, Figure S1: Identification of potentially AtfA and AtfB dependent genes; Figure S2: Principal component (PC) analysis of the transcriptomes; Figure S3: Transcriptional profile of *easB* encoding the polyketide synthase of the Emericellamide secondary metabolite cluster in mycelial samples; Table S1: Lists and characteristics of AtfA- and AtfB-dependent genes; Table S2: List, characteristics and RPKM values of genes that showed both AtfA- and AtfB-dependence in mycelial and conidial samples; Table S3: Results of the gene set enrichment analyses for AtfA- and AtfB-dependent genes; Table S4: Characterization of AtfA- and AtfB-dependent genes by their known or putative functions.
