# Supplementary material for: Genome-Wide Gene Expression Analyses of the AtfA/AtfB-Mediated Menadione Stress Response in Aspergillus nidulans
Source: Cells. 2023 Jan 31;12(3):463. doi: 10.3390/cells12030463 (PMC9913763; doi:10.3390/cells12030463)
Supplement: Supplementary file 1 [file cells-12-00463-s001.zip › Supplementary Materials.pdf]

Supplementary materials

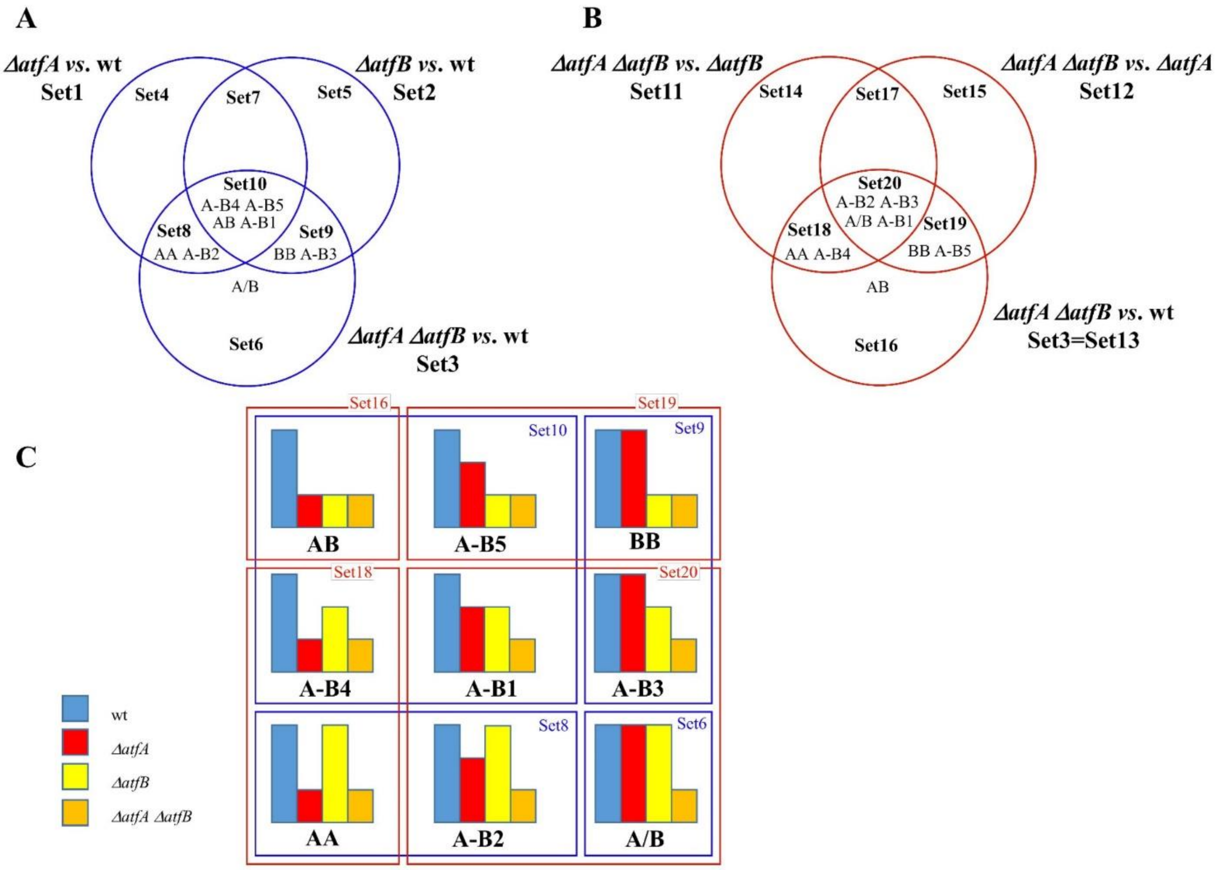

Figure S1. Identification of potentially AtfA and AtfB dependent genes. (**A**,**B**): Comparisons and gene sets used to identify potentially AtfA and AtfB dependent genes. (**C**): Expected transcriptional behaviour of genes in the marked subsets. Blue (control), red (*ΔatfA*), yellow (*ΔatfB*) and orange (*ΔatfA ΔatfB*) rectangles with different length represent significantly different transcriptional activity of genes in different strains.

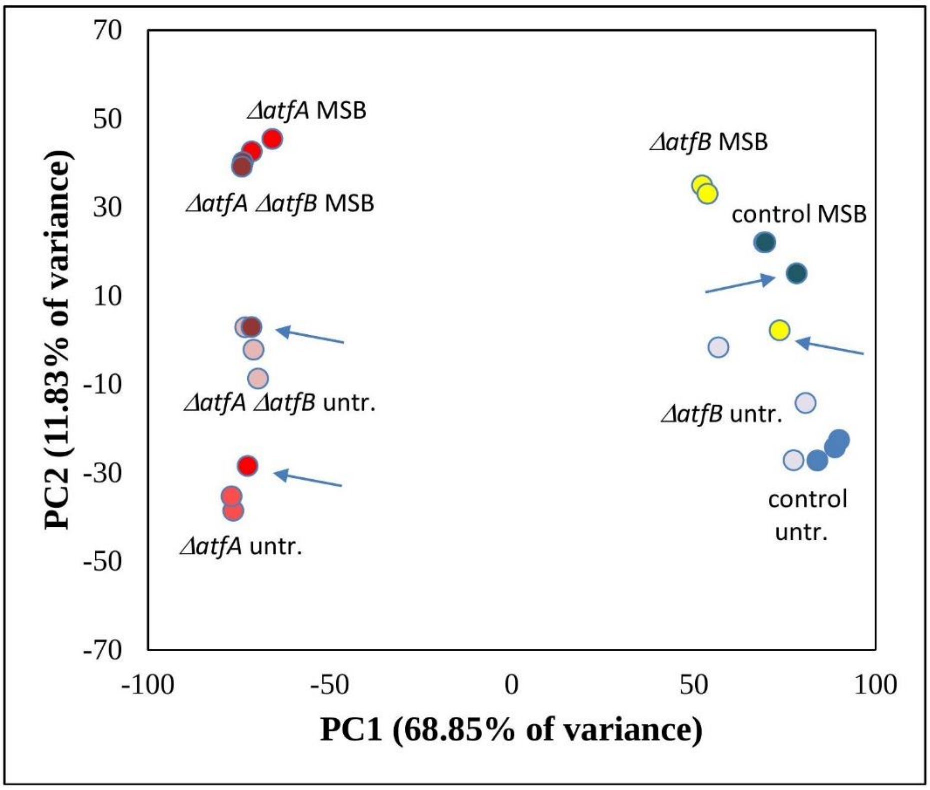

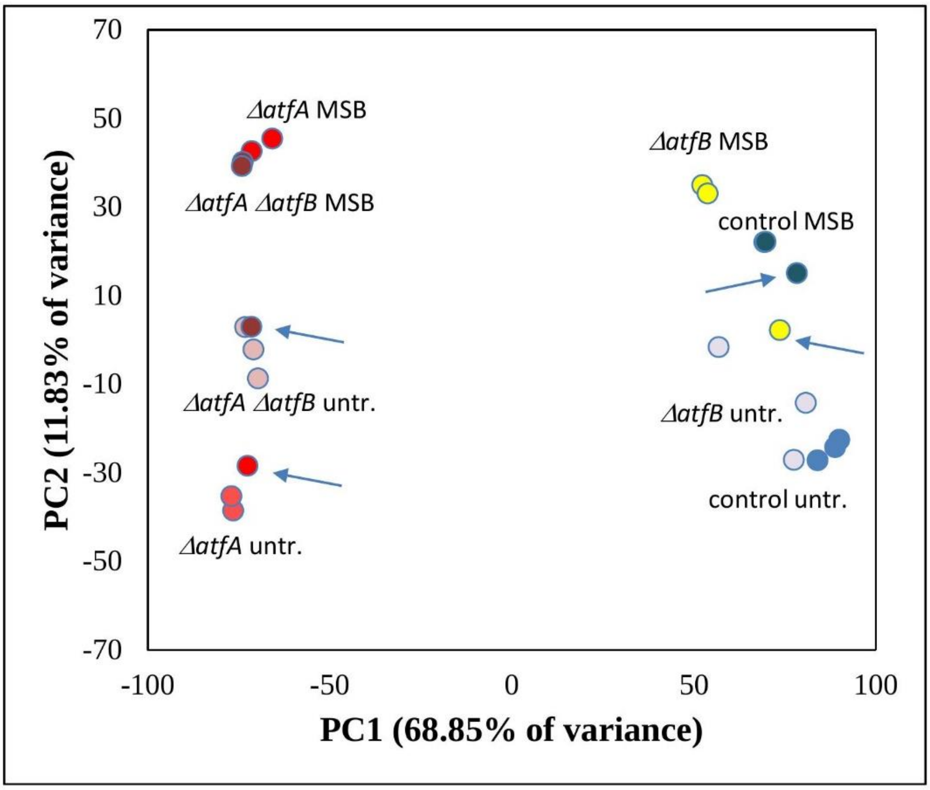

Figure S2 Principal component (PC) analysis of the transcriptomes.
PC analysis was carried out using the rlog data generated with the DeSeq2 software. (**A**) mycelial samples; (**B**) conidial samples; Arrows indicate the conidial samples obtained in separate experiments.

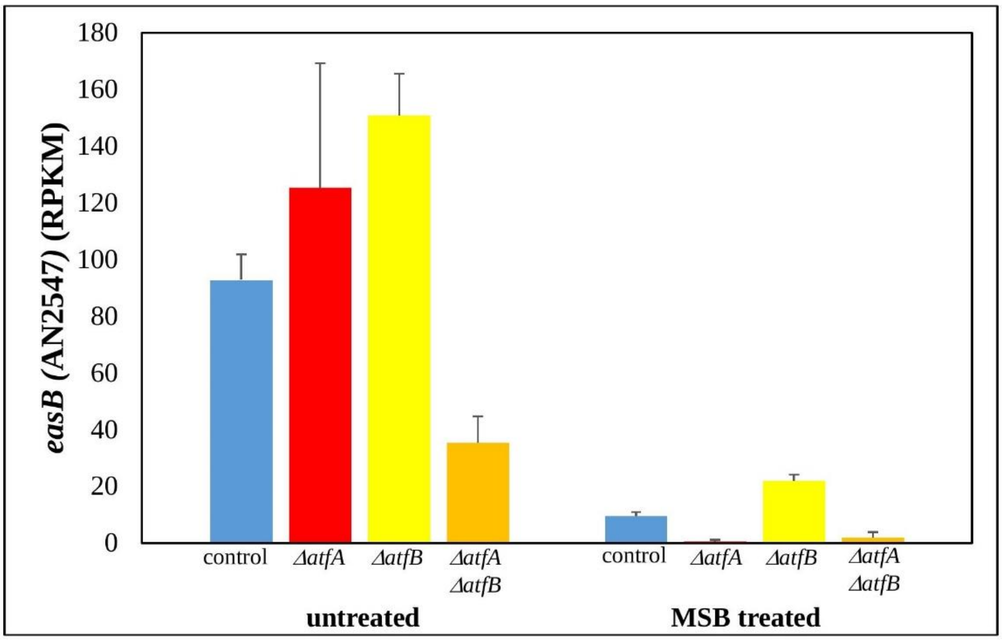

Figure S3 Transcriptional profile of *easB* encoding the polyketide synthase of the Emericellamide secondary metabolite cluster in mycelial samples. Mean ± SD RPKM values calculated from the three biological replicates are presented.

Table S1 Lists and characteristics of AtfA- and AtfB-dependent genes.
down - downregulated by MSB stress treatment
up - upregulated by MSB stress treatment
AA - regulated by AtfA but not by AtfB
BB - regulated by AtfB but not by AtfA
AB - Regulated by both AtfA and AtfB; deletion of either *atfA* or *atfB* reduces the gene activity at least to the level of the double mutant.
A/B - Regulated by both AtfA and AtfB; deletion of only *atfA* or only *atfB* does not reduce the gene activity.
A-B - Regulated by both AtfA and AtfB; deletion of *atfA* or *atfB* reduces only partially the gene activity. (See also Fig. S1)

Table S2 List, characteristics and RPKM values of genes that showed both AtfA- and AtfB-dependence in mycelial and conidial samples.

Table S3 Results of the gene set enrichment analyses for AtfA- and AtfB-dependent genes.
Sheet 1 AtfA-dependent genes (Set26), untreated cultures, mycelium
Sheet 2 AtfA-dependent genes (Set26), MSB-treated cultures, mycelium
Sheet 3 AtfA-dependent genes (Set26), untreated cultures, conidium
Sheet 4 AtfA-dependent genes (Set26), MSB-treated cultures, conidium

Sheet 5 AtfB-dependent genes (Set27), untreated cultures, mycelium
Sheet 6 AtfB-dependent genes (Set27), MSB-treated cultures, mycelium
Sheet 7 AtfB-dependent genes (Set27), untreated cultures, conidium
Sheet 8 AtfB-dependent genes (Set27), MSB-treated cultures, conidium

Table S4 Characterization of AtfA- and AtfB-dependent genes by their known or putative functions.
Sheet 1 Secondary metabolite cluster genes
Sheet 2 Selected carbohydrate metabolism gene groups
Sheet 3 Antioxidative enzyme, Fe-S cluster assembly, phosphorelay response regulator and transcription factor genes
Sheet 4 TCA cycle and respiration genes
